# Effects of Acrylamide-Induced Vasorelaxation and Neuromuscular Blockage: A Rodent Study

**DOI:** 10.3390/toxics9060117

**Published:** 2021-05-24

**Authors:** Wei-De Lin, Chu-Chyn Ou, Shih-Hao Hsiao, Chih-Han Chang, Fuu-Jen Tsai, Jiunn-Wang Liao, Yng-Tay Chen

**Affiliations:** 1 Department of Medical Research, Human Genetic Center, School of Post-Baccalaureate Chinese Medicine, China Medical University Hospital, Taichung 404, Taiwan; weide@mail.cmu.edu.tw; 2 School of Nutrition, Chung Shan Medical University Hospital, Taichung 402, Taiwan; occ@csmu.edu.tw; 3 Graduate Institute of Food Safety, National Chung Hsing University, Taichung 402, Taiwan; howardcartw@live.com; 4 Bachelor Program of Biotechnology, National Chung Hsing University, Taichung 402, Taiwan; s107030313@mail.nchu.edu.tw; 5 Department of Medical Research, Human Genetic Center, School of Chinese Medicine, China Medical University Hospital, Taichung 404, Taiwan; d0704@mail.cmuh.org.tw; 6 Department of Biotechnology and Bioinformatics, Asia University, Taichung 413, Taiwan; 7 Graduate Institute of Veterinary Pathobiology, National Chung Hsing University, Taichung 402, Taiwan

**Keywords:** acrylamide, vasorelaxation, neurotoxicity, nitric oxide synthase, nicotinic acetylcholine receptor

## Abstract

Acrylamide (ACR), which is formed during the Maillard reaction, is used in various industrial processes. ACR accumulation in humans and laboratory animals results in genotoxicity, carcinogenicity, neurotoxicity, and reproductive toxicity. In this study, we investigated the mechanisms by which ACR may induce vasorelaxation and neuromuscular toxicity. Vasorelaxation was studied using an isolated rat aortic ring model. The aortic rings were divided into the following groups: with or without endothelium, with nitric oxide synthase (NOS) inhibition, with acetylcholine receptor inhibition, and with extracellular calcium inhibition. Changes in tension were used to indicate vasorelaxation. Neuromuscular toxicity was assessed using a phrenic nerve–diaphragm model. Changes in muscle contraction stimulated by the phrenic nerve were used to indicate neuromuscular toxicity. ACR induced the vasorelaxation of phenylephrine-precontracted aortic rings, which could be significantly attenuated by NOS inhibitors. The results of the phrenic nerve–diaphragm experiments revealed that ACR reduced muscle stimulation and contraction through nicotinic acetylcholine receptor (AChR). ACR-induced vasotoxicity was regulated by NOS through the aortic endothelium. Nicotinic AChR regulated ACR-induced neuromuscular blockage.

## 1. Introduction

Humans are exposed to acrylamide (ACR) from different sources, including various foodstuffs such as French fries, potato chips, and bread; cigarette smoke; and industrial ACR production [1]. The oral lethal dose (LD_50_) of ACR is 107–251 mg/kg for rats, 150–180 mg/kg for guinea pigs, and 107–170 mg/kg for mice [2]. Owing to its small molecular weight, ACR can be absorbed through the skin (LD_50_: 400 mg/kg in rats) [3]. ACR induces reproductive toxicity, genotoxicity, neurotoxicity, and carcinogenicity [4,5,6,7]. In rats, ACR intoxication can induce lipid peroxidation and nitric oxide secretion and reduce glutathione, glutathione peroxidase, and superoxide dismutase levels in the brain, liver, and kidneys [8,9,10].

The level of urinary ACR and its metabolites in the early stages of paediatric chronic kidney disease may identify patients at risk of cardiovascular disease (CVD) [11]. ACR haemoglobin biomarkers are significantly associated with CVD in active smokers and individuals exposed to environmental tobacco smoke but not in nonsmokers [12]. ACR induces the constriction of the vascular canals of primary osteons in mice and causes aortic ring contraction in rats [13], thus reducing the luminal area of rat aortas. ACR also induces responses to phenylephrine (PE) and potassium chloride (KCl) [13] and attenuates endothelium-dependent artery relaxant response to acetylcholine in rabbits [14].

ACR is toxic to the nervous systems of both humans and animals [15]. It causes swelling at the ends of axons of both the central and peripheral nervous systems and induces paralysis of the cerebrospinal system in humans [16]. Early symptoms of these neurological disorders include muscle weakness, difficulty in walking, numbness in the limbs, decreased tactile sensation, and disappearance of tendon reflexes. Patients with severe poisoning experience tremors, ataxia, unconsciousness, dizziness, memory loss, and delusions [17]. ACR also causes nerve palsy and dyskinesia as well as damage to skeletal muscles, cardiac muscles, and the small intestine [18].

Dietary acrylamide intake may increase the risks of some cancer types [19,20,21]. However, patterns of increased risks of various cancers in the rat model have not been observed in epidemiological studies, indicating that the rodent model may not be appropriate for ACR cancer research. [22]. ACR is also toxic to the reproductive system in animal models [23].

How ACR causes vasorelaxation and neuromuscular toxicity remains unclear. This study investigated the mechanisms underlying ACR-induced vasorelaxation and neuromuscular toxicity by using a rat aortic ring model and a mouse phrenic nerve–diaphragm model, respectively. We followed the ARRIVE guidelines for reporting on animal studies [24,25,26]. In this study, the isolated rat aortic ring model was used due to its relevance to ACR related to cardiovascular toxicity, and the phrenic nerve–diaphragm model was used due to its relevance to ACR-induced muscle stretching (paralysis) research. The primary advantage of these technologies is that they use living tissue that functions as a whole tissue, with a physiological outcome (contraction or relaxation) that is relevant to the body. It involves a series of steps: drug–receptor interaction, signal transduction, second messenger generation, change in smooth muscle excitability, and change in tissue function. Although other techniques allow the study of each of these steps (e.g., radioligand binding for drug affinity and measurement of second messengers), the isolated tissue bath technique permits the integration of all these steps. Another advantage is that retaining tissue function facilitates the calculation of critical pharmacological variables that are more meaningful in a tissue versus a cellular setting; this is closer to how the drugs work in the body as a whole [27].

## 2. Materials and Methods

### 2.1. Chemicals

ACR, ethylene glycol tetra-acetic acid (EGTA), atropine, mecamylamine hydrochloride, Nω-Nitro-L-arginine methyl ester hydrochloride (L-NAME), nereistoxin (NTX), and phenylephrine (PE) were purchased from Sigma-Aldrich (Saint Louis, MO, USA). Sodium chloride, KCl, calcium chloride dehydrate, potassium dihydrogen phosphate, magnesium sulphate heptahydrate, sodium hydrogen carbonate, and glucose were purchased from Merck (Darmstadt, Germany).

### 2.2. Animals

The rat aorta is a good ex vivo model for a vasotoxicity assay, and the mouse phrenic nerve–diaphragm model is a well-established ex vivo model for a neuromuscular toxicity assay. We used these two animal models in this study. Male Sprague Dawley (SD) rats, weighing 232.6 ± 11.2 g, and male Institute of Cancer (ICR) mice, weighing 27.6 ± 1.5 g, were obtained from Bio LASCO Taiwan (I-Lan, Taiwan). Because the tissue bath experiments used dissected tissue from rodents and the effects of hormones were negligible, we selected male rats and mice for our study. The animals were housed in individual cages and fed lab chow ad libitum (Lab DietR 5001 Rodent diet; PMIR Nutrition International, St. Louis, MO, USA), and they were provided deionised reverse-osmosis water ad libitum. The animals were housed at 20–25 °C and 40–70% relative humidity under a photoperiod 12-h light/dark cycle. The experiments were fulfilled according to the Guide for the Care and Use of Laboratory Animals. All protocols were approved by the Institutional Animal Care and Use Committee of National Chung Hsing University, Taiwan (IACUC: 107-114, 2019).

### 2.3. Assessment of Vasorelaxation

#### 2.3.1. Rat Aortic Ring Preparation

**Figure 1:**
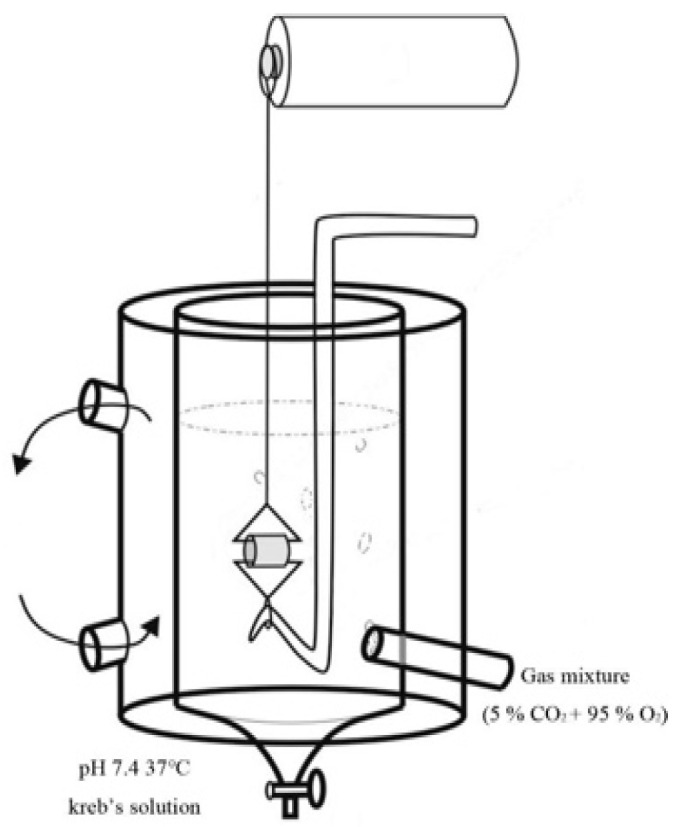
Rat aortic ring in an organ bath.

The rat aortic rings were isolated according to previously described methods [28]. The rats were anaesthetised with an intraperitoneal injection of urethane (0.6 g/kg body weight) combined with chlorohydrate (0.4 g/kg body weight). Thereafter, blood was exsanguinated from the abdominal aorta. The thoracic aorta was removed and separated in normal Krebs solution (118.5 mM NaCl, 4.8 mM KCl, 2.5 mM CaCl_2_ 2H_2_O, 1.2 mM KH_2_PO_4_, 1.2 mM MgSO_4_ 7H_2_O, 25 mM NaHCO_3_, 11.1 mM glucose). The aorta was cut into 6–8-mm-long rings and subjected to an organ bath perfused with 95% O_2_ and 5% CO_2_ at 37 °C. Two “L”-type stainless steel hooks were then inserted into the aortic lumen, with one fixed to the bottom and the other connected to a force transducer. The aortic rings were maintained under the optimal tension of 1 g for 45 min (Figure 1). Aortic ring contraction was recorded using a force-displacement transducer (Grass SD9 stimulator; Quincy, MA, USA) that was connected to a PowerLab recorder (ADIntruments, New South Wales, Australia). The aorta rings were denuded and the endothelium was removed by rubbing with cotton; the absence of ACh-induced relaxation indicated successful denudation [28].

#### 2.3.2. Dosage Selection

The no-observed-adverse-effect level for ACR is 0.3–1.0 nmol/g globin for mild reversible symptoms of the peripheral nervous system. Severe neurological symptoms are observed at an exposure level of over 20 nmol/g globins, corresponding to an estimated ACR intake of approximately 0.8 mg/kg body weight [29].

#### 2.3.3. Effects of ACR on Rat Aortic Rings

Forty male SD rats were randomly divided into eight groups of five rats each. We used 2 mM ACR as the initial experimental dosage, which was within the range measured in humans. In the vasorelaxation study, the aorta rings were precontracted with PE (2 µM) for 15 min, and ACR (0, 2, 4, and 8 mM) was added to the endothelium-intact and endothelium-denuded aortic rings in an organ bath. The samples were individually incubated for 60 min. The percentage of ACR-induced relaxation in PE-contracted aortic rings was calculated after ACR incubation.

#### 2.3.4. Effects of Nitric Oxide Synthase and AChR Inhibitors on the ACR-Relaxed Aortic Ring of Rats

Twenty male SD rats were randomly divided into four groups of five rats each. The aorta rings were precontracted using PE (2 µM) for 15 min and preincubated with L-NAME (a nitric oxide synthase [NOS] inhibitor, 100 µM), atropine (a muscarinic acetamidine choline receptor inhibitor, 50 µM), and mecamylamine (a nicotinic AChR inhibitor, 10 µM) for 10 min. Next, 2 mM ACR was added to the aortic rings, and the samples were individually incubated for 60 min.

#### 2.3.5. Effects of ACR-Induced Relaxation with/without Ca^2+^

Ten male SD rats were randomly divided into two groups of five rats each. Endothelium-denuded aortic rings were incubated with either Krebs solution or a Ca^2+^-free modified Krebs solution (with 50 µM EGTA) to mimic the in vivo environment. The aortic rings were precontracted using PE (5 µM) for preconstruction. Subsequently, ACR was added for 15 min, followed by CaCl_2_ (5 mM). The relaxation of aortic rings was measured for 60 min, and ACR-induced relaxation was expressed as a percentage of PE-induced maximal contraction.

### 2.4. Assessment of Neuromuscular Toxicity

#### 2.4.1. Preparation and Treatment of the Phrenic Nerve–Diaphragm Model

**Figure 2:**
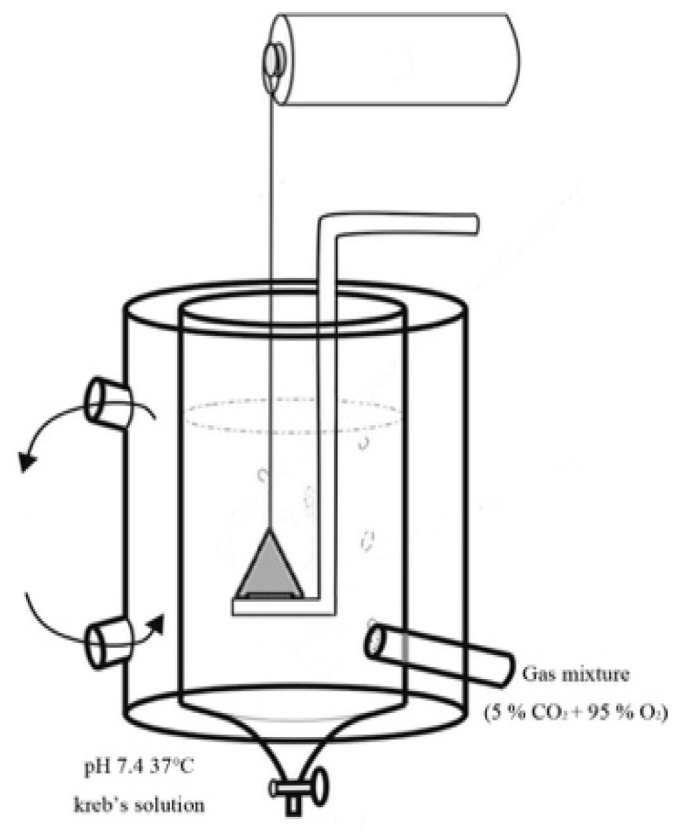
Mouse phrenic nerve-diaphragms in an organ bath.

The phrenic nerve–diaphragms were isolated as described previously [30,31]. The mice were killed with CO_2_ exposure. Using a pair of forceps, a cotton thread was inserted under the nerve, and two knots were tied. The diaphragm was held using the lower part of the sternum to avoid damaging the muscle fibres. It was then transferred to normal Krebs solution maintained under 95% O_2_ and 5% CO_2_ at 37 °C. A cotton thread was tied (with double knots) at the apex of the triangle to connect the preparation to the transducer (Grass SD9 stimulator; Quincy, MA, USA), which was connected to a PowerLab recorder (ADIntruments, Bella Vista, NSW, Australia). The ribs at the base of the triangle were used to secure the preparation to the support. Next, the tissue was drilled between the ribs, a cotton thread was passed through, and a double knot was tied around the lower rib. The support was positioned, and the base of the triangle was tied using a double knot around the holder (Figure 2). Muscle twitches were evoked by indirect stimulation of the phrenic nerve for 0.05 ms at 0.2 Hz or by direct stimulation of the muscle with a pulse for 0.5 ms at 0.2 Hz. The phrenic nerve–diaphragms were equilibrated in Krebs solution and maintained under the optimal tension of 1 g for 45 min before the test.

#### 2.4.2. Effects of ACR on Phrenic Nerve–Diaphragm in Mice

Twenty male ICR mice were randomly divided into four groups of five mice each. To determine the effect of ACR on the neuromuscular junction, ACR (0, 2, 4, and 8 mM) was added to the organ bath containing isolated mouse phrenic nerve–diaphragms. Both nerve- and muscle-evoked twitches were continually recorded for 80 min. For this experiment, 120 min is a commonly used time for the dissected phrenic nerve–diaphragm to stay in the organ bath.

#### 2.4.3. Effects of AChR Inhibitor on ACR-Induced Phrenic Nerve–Diaphragm Changes

Ten male ICR mice were randomly divided into two groups of five mice each. To assess the effects of neuromuscular blockage on ACR-induced changes, the isolated mouse phrenic nerve–diaphragm was pretreated with 1 mM NTX (a nicotinic AChR inhibitor) for 15 min. After the nerve- or muscle-evoked twitches were inhibited, 8 mM ACR was added.

#### 2.4.4. Effects of Extracellular Ca on ACR-Induced Phrenic Nerve–Diaphragm Changes

Ten male ICR mice were randomly divided into two groups of five mice each. For the Ca^2+^-free experiment, the phrenic nerve–diaphragms were incubated with either Krebs solution or a Ca^2+^-free (with 50 µM EGTA) modified Krebs solution. When the tension returned to the baseline level, 8 mM ACR was added.

### 2.5. Statistical Analysis

The half-effective concentration (EC_50_) was calculated 10 min after ACR treatment using Excel (Microsoft). A one-way analysis of variance was used to analyse the differences between the control and other groups and between different ACR concentrations in the 10 min vasorelaxation results. Tukey’s multiple comparison test was used to determine significance. *p* < 0.05 was considered significant.

## 3. Results

### 3.1. ACR-Induced Aortic Ring Vasorelaxation

**Figure 3:**
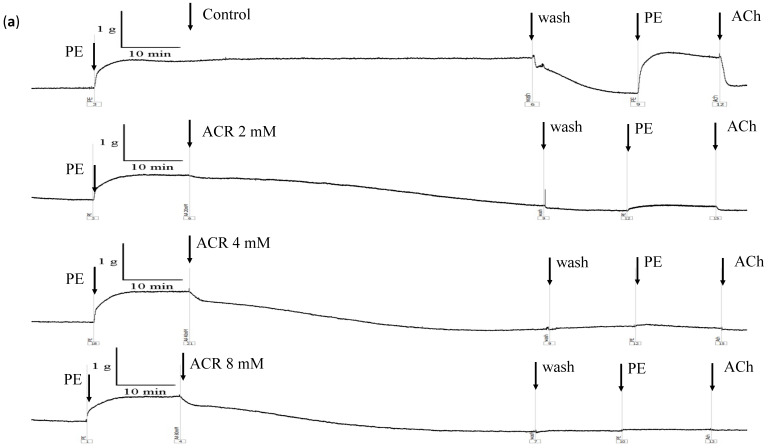

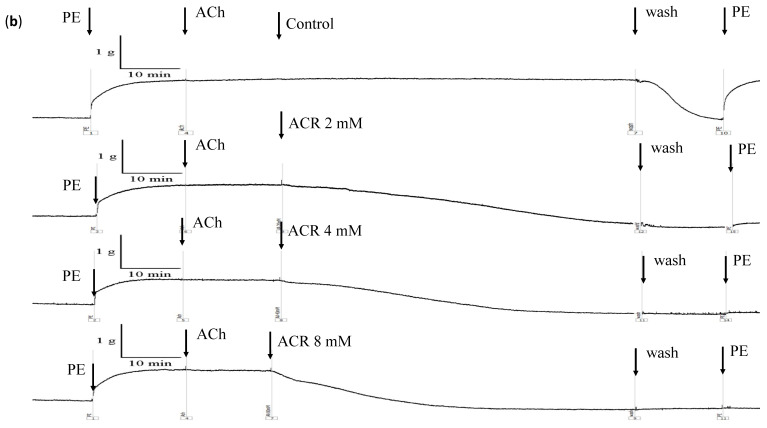

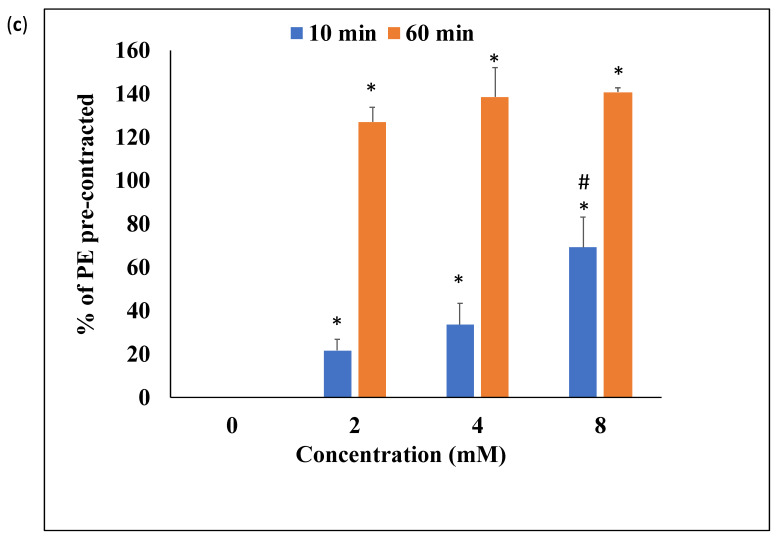

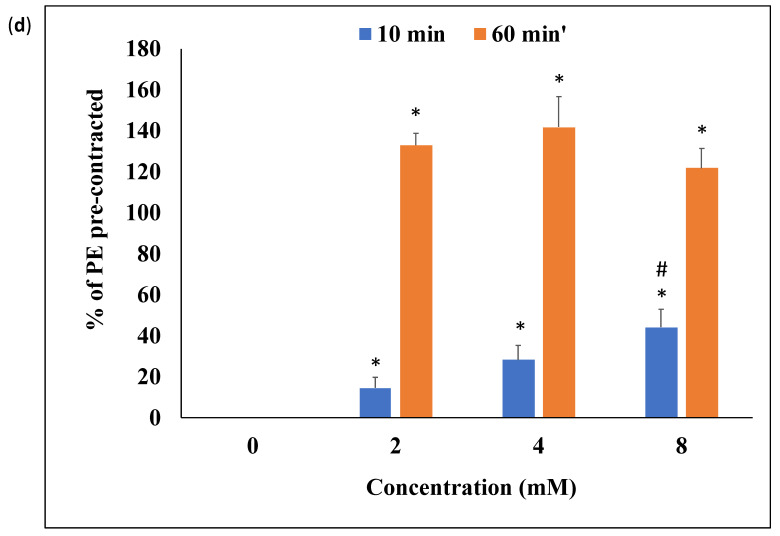
Effects of acrylamide (ACR) on endothelium-intact (**a**,**c**) and endothelium-denuded (**b**,**d**) aortic rings. Aortic rings were precontracted using phenylephrine (PE) (2 μM) for 15 min, and the control (ACR 2, 4, and 8 mM) was added to the aortic rings. The samples were individually recorded for 60 min. (*: significant difference vs. control, *p* < 0.05; #: significant difference between ACR 4 mM and ACR 8 mM).

When the endothelium-intact or -denuded groups were treated with ACR (0, 2, 4, and 8 mM), the contractile tension induced by PE decreased in a concentration-dependent manner (Figure 3a,b). By contrast, the contractile tension did not decrease with time in the control group. After 10 min of treatment with ACR at concentrations of 0, 2, 4, and 8 mM, the contractile tension decreased by 0%, 21.7% ± 5.1%, 33.6% ± 9.8%, and 69.1% ± 14.0%, respectively (Figure 3c), in the endothelium-intact group and 0%, 14.6% ± 5.1%, 28.1% ± 7.2%, and 44.2% ± 8.7%, respectively (Figure 3d), in the endothelium-denuded group. The EC_50_ values of ACR-induced relaxations in the endothelium-intact and -denuded aortic rings were 5.75 and 8.53 mM, respectively. After 60 min of treatment with ACR at concentrations of 0, 2, 4, and 8 mM, the contractile tension decreased by 0%, 127.0% ± 6.7%, 138.5% ± 13.5%, and 140.8% ± 1.9%, respectively (Figure 3c), in the endothelium-intact group and 0%, 132.9% ± 5.8%, 141.8% ± 14.8%, and 121.6% ± 9.7%, respectively (Figure 3d), in the endothelium-denuded group. After observing the endothelium-intact or -denuded group for 60 min, the aortic ring was flushed with a physiological buffer solution without ACR. After recontraction with PE, the contractile tension of the aortic ring in the 0-, 2-, 4-, and 8-mM treatment groups was 103.9% ± 23.9%, 11.4% ± 6.3%, 8.4% ± 9.3%, and 1.3% ± 2.9%, respectively (intact group), and 89.5% ± 22.5%, 14.4% ± 4.0%, 6.3% ± 2.6%, and 0.8% ± 0.6%, respectively (denuded group). Thus, ACR-induced vasorelaxation occurred in a concentration-dependent manner in both the endothelium-intact and -denuded aortic ring groups.

### 3.2. Effects of L-NAME, Atropine, and Mecamylamine on ACR-Induced Vasorelaxation

The basic functioning of endothelium-intact aortic rings was verified after a 10-min relaxation rate of >20% was observed. After L-NAME treatment, the 10-min relaxation rate of endothelium-intact aortic rings was reduced to <5% (Table 1). Moreover, ACR-induced relaxation could be significantly attenuated by adding a NOS inhibitor. ACh is an essential factor in endothelial-dependent vasorelaxation. AChR is considered the main receptor involved in inducing vasorelaxation. Endothelium-intact aortic rings were pretreated with atropine, a muscarinic AChR inhibitor. The 10-min relaxation rate in the atropine pretreatment group was 37.6% ± 7.4%. A significant difference was observed between the two groups (Table 1, *p* < 0.05). Atropine did not inhibit the vasorelaxation induced by ACR, and the degree of vasorelaxation was exacerbated. However, the 60-min relaxation rate in the atropine pretreatment group decreased by 138.4% ± 15.4%. No significant intergroup difference was observed. Mecamylamine is an inhibitor of nicotinic AChR. After pretreatment with mecamylamine, the 10-min relaxation rate in the mecamylamine pretreatment group was 19.7% ± 7.0% (Table 1), and no significant between-group difference was observed. However, although the 60-min relaxation rate in the mecamylamine pretreatment group was 96.8% ± 1.8%, the decrease in the control group was 127.0% ± 6.7%, and the difference was significant (Table 1, *p* < 0.05). A significant difference was observed between the L-NAME and mecamylamine treatment groups at 10 min (*p* < 0.05), but not at 60 min.

### 3.3. Effects of Ca^2+^ on ACR-Induced Vasorelaxation

Ca^2+^ is an essential factor in smooth muscle contraction and relaxation. We examined the effect of ACR when extracellular CaCl_2_ (5 mM) was added. At 10 min after the addition of Ca^2+^ ions, an increase in contraction was observed in the experimental group by 231.5% ± 60.4% and in the control group (only ACR treatment) by 170.0% ± 38.2% (Table 2; *p* > 0.05). At 60 min, the treatment group exhibited a relaxation of −136.1% ± 49.7%, whereas the control group exhibited a contraction of 226.9% ± 53.3% (Table 2; *p* < 0.05). Thus, the experimental group was affected by ACR-induced vasorelaxation.

### 3.4. Effects of ACR on Isolated Mouse Phrenic Nerve–Diaphragm

**Figure 4:**
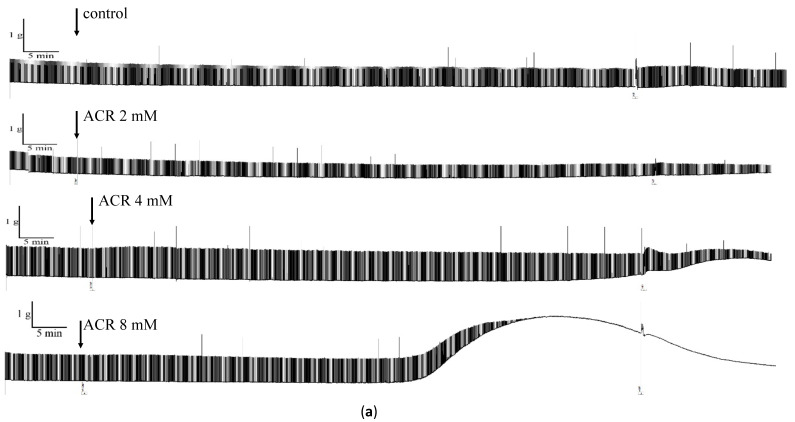

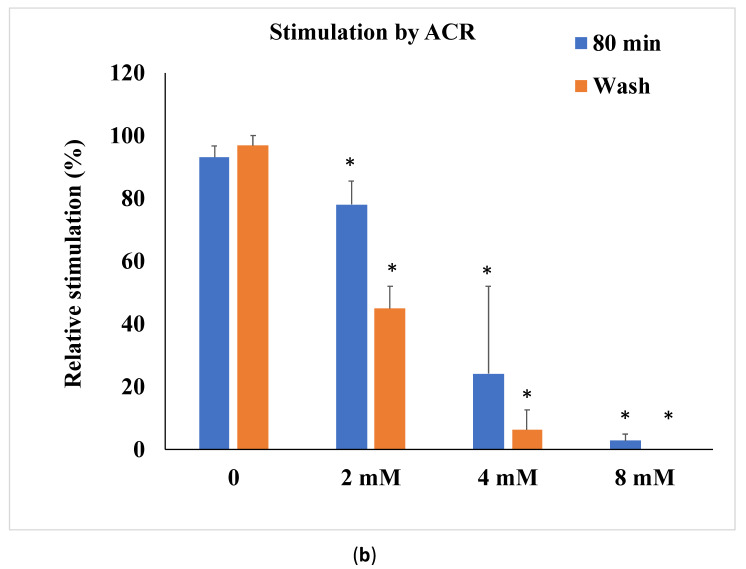

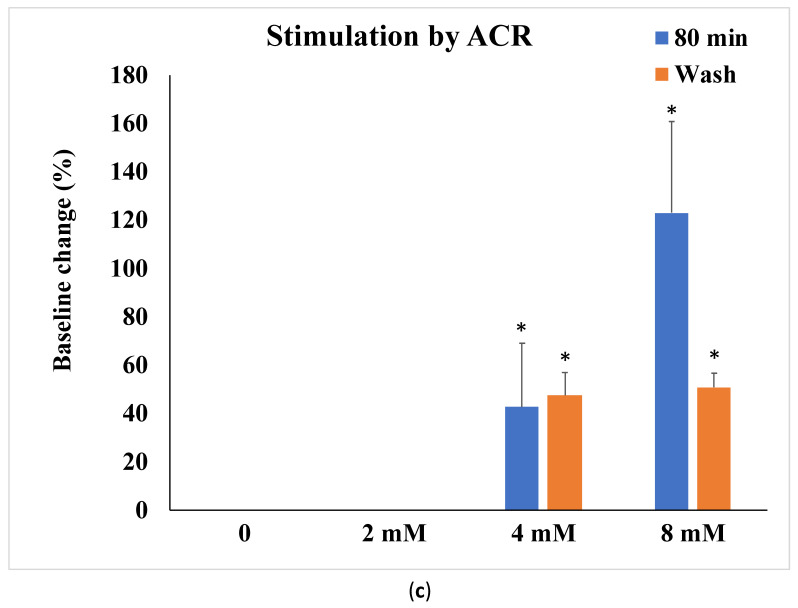
Effects of acrylamide (ACR) on isolated mouse phrenic nerve–diaphragm. (**a**) The organ baths were treated with 0, 2, 4, and 8 mM ACR for 80 min. After 80 min of ACR treatment, samples of mouse phrenic nerve–diaphragm were refreshed with physiological buffer solution. Effect of ACR on the isolated mouse phrenic nerve–diaphragm (**b**) neuromuscular blocking and (**c**) muscle contraction. Data are expressed as means ± standard deviations (*n* = 5) (* *p* < 0.05 vs. control).

Relative stimulation rate in the ACR-treated group:

The relative tension rates of groups treated with 0, 2, 4, and 8 mM ACR for 80 min were 93.1% ± 3.6%, 78.1% ± 7.4%, 24.2% ± 27.8%, and 2.7% ± 2.2%, respectively (Figure 4a,b), indicating a dose-dependent decrease (*p* < 0.05). Next, the samples were rinsed with a physiological buffer solution containing no ACR; the relative stimulation rates were 96.8% ± 3.2%, 44.9% ± 7.1%, 6.3% ± 6.3%, and 0% in the 0-, 2-, 4-, and 8-mM experimental groups, respectively, exhibiting a dose-dependent decrease (*p* < 0.05) (Figure 4a,b). Therefore, after 80 min of ACR treatment (at 2, 4, and 8 mM), the mouse phrenic nerve–diaphragm samples did not return to their original beat amplitude after being rinsed with fresh physiological buffer solution (*p* < 0.01).

Degree of muscle contraction in the ACR-treated group:

The muscle contraction rates of groups after treatment with 0, 2, 4, and 8 mM ACR for 80 min were 0%, 0%, 42.5% ± 26.5%, and 123.0% ± 37.7%, respectively (Figure 4c), indicating a dose-dependent increase (*p* < 0.05). After the samples were rinsed with a physiological buffer solution without ACR, the muscle contraction rates were 0%, 0%, 47.4% ± 9.5%, and 50.8% ± 5.8% in the 0-, 2-, 4-, and 8-mM treatment groups, respectively, indicating a dose-dependent increase (*p* < 0.05) (Figure 4c).

### 3.5. Effects of NTX on ACR-Induced Phrenic Nerve Diaphragm Contraction

**Figure 5:**
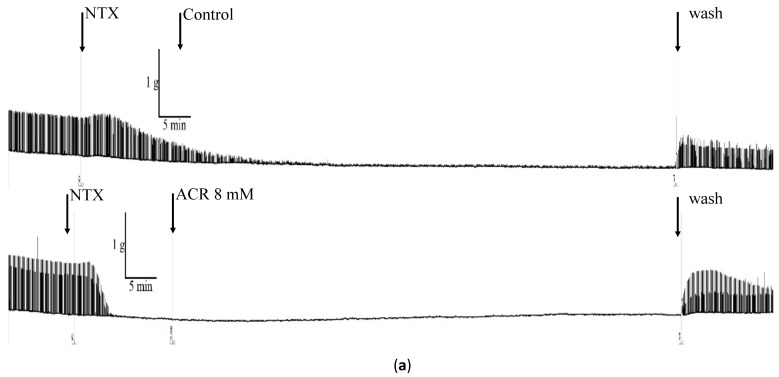

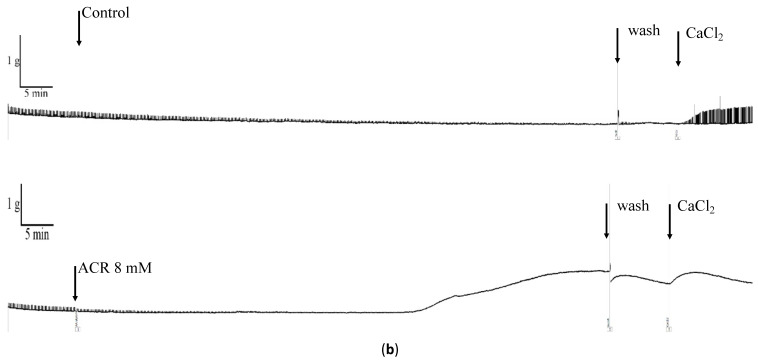
Effect of AChR inhibitor and Ca-chelating agent on isolated mouse phrenic nerve–diaphragm. (**a**) Effects of AChR inhibitor (NTX) on isolated mouse phrenic nerve–diaphragm. The organ baths were pretreated with NTX (a nicotinic AChR inhibitor) for 15 min. Thereafter, 8 mM ACR was added after nerve- or muscle-evoked twitches were observed, and the mouse phrenic nerve–diaphragm was incubated for 80 min. After 80 min of ACR treatment, samples of mouse phrenic nerve–diaphragm were refreshed with physiological buffer solution. Control: only NTX treatment; NTX and ACR treatment. (**b**) Effects of calcium-chelating agent on isolated mouse phrenic nerve–diaphragm. The phrenic nerve–diaphragm baths were incubated with either Krebs solution or a Ca^2+^-free (with 50 µM EGTA) modified Krebs solution. Thereafter, 8 mM ACR was added after the tension returned to the baseline level. Control: only EGTA treatment; EGTA and ACR treatment.

In this experiment, NTX was used to block nerve–muscle transmission, and then the effect of NTX on the phrenic nerve transverse diaphragm was explored (Figure 5a). The control group was pretreated with NTX only. The experimental group was pretreated with NTX, followed by 8 mM ACR. The relative stimulation rate decreased to 9.1% ± 5.1% in the control group and to 0.5% ± 0.8% the experimental group (Table 3; *p* < 0.05). After rinsing with fresh physiological buffer solution, the relative stimulation rate of the control group was 76.7% ± 11.6% and of the experimental group was 62.0% ± 9.8%, indicating their return to normal levels after the rinsing.

We examined the effects of NTX on ACR-induced muscle contraction. The 80-min baseline values were compared with the ACR treatment baseline values. The baseline change in the control group was −19.4% ± 4.2% and in the experimental group was −4.3% ± 6.7% (Table 3; *p* < 0.05). After rinsing with fresh physiological buffer solution, no significant difference in baseline changes was observed (Table 3), indicating that NTX does not cause a change in the baseline.

### 3.6. Effects of Extracellular Ca^2+^ on ACR-Induced Changes in Phrenic Nerve–Diaphragm

We explored the mechanism of muscle contraction by removing Ca^2+^ using EGTA (Figure 5B). The baseline change in the control group was −16.9% ± 3.2% and that in the ACR treatment group was 123.8% ± 86.7%, and the difference was not significant (Table 4). The control group exhibited normal contractions after being rinsed in a solution containing Ca^2+^, but the contraction in the ACR treatment group did not recover (Figure 5B). Therefore, the removal of extracellular Ca^2+^ did not prevent muscle contraction.

## 4. Discussion

In the present study, ACR resulted in a dose-dependent vasorelaxation of rat aortic rings. The EC_50_ values for ACR-induced vasorelaxation in the endothelium-intact and -denuded aortic rings were 5.75 and 8.53 mM, respectively, without significant difference. ACR haemoglobin adduct levels have been reported to be as high as 1.55 nmol/g in ACR production plant workers in the United Kingdom [32], 17.7 nmol/g in tunnel workers in Sweden [33], and 13.4 nmol/g in ACR production workers in China, and according to Paulsson et al., these values corresponded to an in vivo dose of 6.0 mM/h during the life span of erythrocytes (17 weeks) [34]. We used 2 mM as the initial experimental dosage based on the dosage measured in ACR production workers in China [34]. In the present study, ACR treatment caused aortic ring relaxation. In a previous study [13], the experimental doses were 2 and 5 mg/kg/day ACR, and the isolated rat aortic ring test was performed 90 days after ACR administration. The results revealed that after precontraction in PE, the two experimental groups treated with ACR presented a higher contraction than the control group did.

ACR in food increases NOS expression in breast epithelial cells [35]. To determine the role of NOS in ACR-induced vasorelaxation, L-NAME pretreatment was used to inhibit NOS activity, which significantly inhibited ACR-induced vasorelaxation. NOS is involved in vasorelaxation, and L-NAME is an antagonist of NOS and potentiates vasodilation [36]. In the AChR inhibitor group, AChR inhibition did not inhibit ACR-induced vasorelaxation. Thus, vasorelaxation induced by ACR is not regulated by AChR. Muscarinic AChR plays a vital role in ACh-induced vasorelaxation [37]. In addition to ACh, bradykinin and physical shear stress on the vascular endothelium affect the regulation of endothelium-dependent vasorelaxation. These three factors induce endothelial-dependent vasorelaxation, activate endothelial NOS, and produce nitric oxide [38]. Bradykinin also plays a role in inflammatory responses by regulating TNF-α and IL-1, which are often used as inflammatory mediators to trigger subsequent inflammatory reactions [39,40]. ACR can cause cell damage by dysregulating TNF-α, IL-1, and iNOS-induced inflammatory responses and through oxidative stress caused by glutathione consumption [41]. Bradykinin induced a transient increase in intracellular Ca^2+^ concentration in astrocyte cells, which was reduced by both nicotine and donepezil. This reduction was inhibited not only by mecamylamine, an nAChR antagonist, but also by PI3K and Akt inhibitors. Together, these results suggest that nAChR stimulation suppresses the inflammatory response induced by bradykinin via the PI3K-Akt pathway in astrocytes [42]. Therefore, bradykinin may also regulate ACR-induced endothelial-dependent vasorelaxation. Our results indicate that mecamylamine reduces long-term vasorelaxation induced by ACR. However, when mecamylamine pretreatment was followed by ACR treatment, the vascular tone decreased, suggesting that nicotinic AChR may not be essential for endothelial-dependent vasorelaxation.

In this study, the phrenic nerve–diaphragm model revealed that ACR can cause neuromuscular blockade and muscle contraction, both of which were significantly inhibited with the AChR inhibitor. Low concentrations of mecamylamine induced the prolonged inhibition of neuronal nAChR, transient inhibition of muscle nAChR, and inhibition of the NMDA receptor. Mecamylamine inhibition of neuronal nAChR is noncompetitive and voltage-dependent [43].

ACR significantly inhibits acetylcholinesterase (AChE) activity in the brain, although AChE activity gradually returns to normal levels after treatment [44,45]. AChE can be characterised into two large groups: asymmetrical (A group) and spherical (G group). In the neuromuscular junction, the A group predominates. In particular, A12 AChE shows the highest expression [46]. After treatment with ACR, the activity of A12 AChE in the muscle tends to decrease with the dose [47]. Because of AChE inhibition, ACh accumulates in the neuromuscular junction and causes muscle depolarisation, resulting in neuromuscular blockage. The mechanism is similar to organophosphorus pesticide poisoning. The process can cause muscle twitching, and at high concentrations, neuromuscular depression, which can lead to respiratory failure [48]. Hagmar et al. (2001) found significant dose–response associations between adducts and peripheral nervous system symptoms, including fatigue, muscle weakness, numbness of the extremities, and other sensory effects, and they reported that 1.0 nmol/g was the neurological threshold dose for tunnel workers [32]. Intracellular Ca^2+^ levels increased significantly in ACR-treated rats [49]. Our results reveal that muscle contraction was still observed after the removal of Ca^2+^; thus, extracellular Ca^2+^ may not be the main cause of muscle contraction. However, Ca^2+^ balance plays a vital role in muscle contraction. The two main sources of Ca^2+^ in cells are extracellular sources and cytoplasmic organelles [50]. Because muscle contraction still occurs after extracellular Ca^2+^ removal, intracellular Ca^2+^ originating from the sarcoplasmic reticulum might play a role.

The level of ACR intake through food is lower than that inhaled by ACR-exposed workers. Furthermore, ACR intake by children from food sources is two or three times higher than that by adults; therefore, ACR-induced toxicities in children may be higher than those in adults [51]. Long-term, low-dose ACR exposure-induced nerve cell damage may cause neurodegenerative diseases, such as Alzheimer’s disease. The mechanism of ACR-induced muscle stretching requires further molecular research.

## 5. Conclusions

To our knowledge, this is the first study to investigate the effects of ACR on vasorelaxation and neuromuscular blockage by using an organ (tissue) bath. ACR-induced vasotoxicity was regulated by NOS through the aortic endothelium. Furthermore, nicotinic AChR regulated ACR-induced neuromuscular blockage. However, the molecular mechanisms of ACR-induced vasorelaxation and neuromuscular blockage need further research.

## Figures and Tables

**Table 1:** Effects of Nω-Nitro-L-arginine methyl ester hydrochloride (L-NAME) on acrylamide-induced relaxation of the endothelium-intact rat aortic ring.

Group	% of PE Precontracted ^1^	
	10 min	60 min
Control	22.8 ± 3.9	127.0 ± 6.7
L-NAME	4.2 ± 1.3 *	114.3 ± 4.2 *
Atropine	37.6 ± 7.4 *	138.4 ± 15.4
Mecamylamine	19.7 ± 7.0	96.8 ± 1.8 *

* *p* < 0.05 vs. the control group; ^1^: Aortic rings were precontracted with PE for 15 min, preincubated with blank, L-NAME, atropine, and mecamylamine for 10 min, and incubated with 2 mM ACR for 60 min. Data are expressed as means ± standard deviations (*n* = 5).

**Table 2:** Effects of calcium on acrylamide (ACR)-induced relaxation of the endothelium-denuded rat aortic ring.

Group	% of PE Precontracted ^1^	
	10 min	60 min
Control	170.0 ± 38.2	226.9 ± 53.3
ACR	231.5 ± 60.4	−136.1 ± 49.7 *

*p < 0.05 vs. the control group; ^1^: The percentage of relaxation of PE-induced contraction by ACR was calculated after 10 or 60 min of CaCl_2_ incubation. Data are expressed as means ± standard deviations (*n* = 5).

**Table 3:** Effect of nereistoxin (NTX) with or without ACR treatment on the isolated mouse phrenic nerve–diaphragm.

	Relative Stimulation (%)		Baseline Change (%)	
	Control	ACR	Control	ACR
80 min	9.1 ± 5.1	0.5 ± 0.8 *	−19.4 ± 4.2	−4.3 ± 6.7 *
wash	76.7 ± 11.6	62.0 ± 9.8	−21.4 ± 5.0	−4.8 ± 8.2 *

* *p* < 0.05 vs. the control group. Data are expressed as mean ± standard deviation (*n* = 5).

**Table 4:** Effect of ethylene glycol tetra-acetic acid with or without acrylamide treatment on the isolated mouse phrenic nerve–diaphragm.

	Baseline Change (%)	
	Control	ACR
80 min	−16.9 ± 3.2	123.8 ± 86.7
Wash	−16.2 ± 3.7	88.3 ± 46.2 *
Ca^2+^	−13.4 ± 3.4	71.8 ± 9.5 *

*: Significant difference between the control and treated groups at *p* < 0.05. Data are expressed as mean ± SD (*n* = 5).
